# Shared Neural Codes for Emotion Recognition in Emoji and Human Faces

**DOI:** 10.1111/psyp.70268

**Published:** 2026-03-02

**Authors:** Madeline Molly Ely, Chloe Kelsey, Géza Gergely Ambrus

**Affiliations:** ^1^ Department of Psychology Bournemouth University Dorset UK

**Keywords:** electroencephalography, emoji, emotions, face processing, facial expressions, multivariate pattern analysis

## Abstract

Facial expressions are critical social signals that support human communication. In digital contexts, emojis serve as a primary surrogate for nonverbal cues such as facial expressions; however, little is known about the extent to which emoji expressions are processed using neural mechanisms similar to those engaged by real human faces. To address this question, we used EEG‐based multivariate pattern analysis (MVPA) to examine the neural dynamics of emotional expression processing in real faces and emoji faces. Across two experiments using identical paradigms, independent groups of participants viewed facial expressions (happy, angry, sad, neutral) in real faces (4 female and 4 male identities, *n* = 24) or emojis (6 platforms, *n* = 25) while performing a two‐alternative forced‐choice emotion recognition task. Time‐resolved multivariate classification and spatio‐temporal searchlight analyses revealed robust decoding of emotional expressions within and across experiments. Consistent effects emerged early and peaked between 145 and 160 ms over posterior‐occipital and parietal regions. Notably, robust cross‐classification between real and emoji faces demonstrated that face‐like emoji stimuli evoke neural responses comparable to those elicited by real faces, with more sustained effects over right posterior sites. These findings suggest that the brain uses partially overlapping spatio‐temporal codes for naturalistic and symbolic facial expressions, providing new insights into the neural coding of social signals and the representational overlap between natural and artificial emotional expressions.

## Impact

Emojis are a primary substitute for facial expressions in digital communication, yet it remains unclear whether the brain processes them as emotional signals in the same way as real faces. Using time‐resolved EEG cross‐classification, this study demonstrates that emotional expressions conveyed by emojis and human faces share overlapping neural representations. These findings advance our understanding of social signal processing across natural and digital contexts and inform theories of emotion perception and human‐computer interaction.

## 1 Introduction

Facial expression recognition is a fundamental aspect of human social interaction, enabling one to infer emotions and intentions from others' facial cues (Frith 2009; Jack and Schyns 2015, 2017; Krumhuber et al. 2023; Schmidt and Cohn 2001). In parallel, the advent of digital communication introduced emojis as established tools for conveying emotions in online interactions (Kaye et al. 2017). These simplified, iconic representations serve as surrogates for facial expressions, raising questions about how the brain processes such abstracted emotional cues compared to real human faces (Kaye et al. 2021). This data‐driven study aims to address the adaptability and generalization of the neural mechanisms involved in emotion recognition by investigating whether real and emoji faces are processed similarly at the neural level.

Previous studies using both mass‐univariate ERP analyses and multivariate decoding have shown that neural sensitivity to human facial expressions emerges rapidly, with emotion‐related information becoming detectable approximately 100 ms after stimulus onset. Mass‐univariate ERP studies have reported emotion‐related discriminations not only at the N170, but also as early as the P1 and across the P1‐N170 and N170‐P2 intervals, with some reports of largest effects in the N170–P2 range; these discriminations can depend on perceived valence and arousal (e.g., Durston and Itier 2021, 2025; Itier and Durston 2023; Mueller et al. 2025). In an EEG classification study, Smith and Smith (2019) reported that both facial expression and identity effects peaked within 90–170 ms over posterior electrodes, with task context influencing identity decoding at early stages but not expression decoding. Similarly, Muukkonen et al. (2020), in an EEG‐fMRI fusion experiment, observed that emotion information became available around 100 ms, spreading from occipital to temporal areas within the first 100–250 ms, with signals for happy faces peaking earlier than those for angry or fearful faces. Using EEG‐based classification, Li, Burton, et al. (2022) found that emotion processing began at approximately 120 ms, preceding identity extraction, which occurred around 235 ms. Additionally, with the help of MEG classification and source estimation, Zhang et al. (2023) demonstrated that regions such as the lateral occipital cortex and inferior parietal cortex differentiated between neutral and expressive faces within the 100–150 ms window, representing categorical information as early as 100 ms.

Recent fMRI studies investigating the neural processing of emojis have largely approached the topic from linguistic or semantic perspectives (Chatzichristos et al. 2020; Dalski et al. 2024). One early fMRI study by Yuasa et al. (2011) explored brain activity related to the emotional valence of kaomoji emoticons (happy or sad). Their findings showed activation in regions such as the right inferior frontal gyrus, cingulate gyrus, and fusiform gyrus, suggesting that abstract emoticons are perceived as dynamic agents, processed similarly to human faces.

Most EEG studies that compare real and emoji faces have concentrated on identifying differences in neural processing between these two stimulus types. Notably, participants tend to recognize emotions expressed by emojis faster and more accurately than those depicted by real faces, which hints at potential differences in how the brain decodes these visual stimuli. For instance, Dalle Nogare and Proverbio (2023) conducted a source‐localized EEG study showing that neural responses to emojis differed significantly from those elicited by human faces. Specifically, while real faces activated the fusiform face area (FFA), emojis elicited N170 components associated with occipital and object‐processing areas. This suggests that the schematic nature of emojis may influence the brain's processing strategies, with the N170 peak occurring earlier for emojis compared to human faces. In addition, the study revealed that both emojis and real faces engaged the limbic system and orbitofrontal cortex, areas related to emotional processing and anthropomorphization.

Further studies have examined different components of the ERP, such as the P100 and LPP, in response to real and emoji faces. Gantiva et al. (2020) found that human faces elicited larger P100 and LPP amplitudes compared to emoji faces, while emojis produced a larger N170 amplitude. Similarly, Yu et al. (2022) investigated emotional violations in facial expressions, emojis, and emotion words, observing classic N400 effects indicative of semantic processing across all stimuli. Notably, the N400 amplitude was smaller for both faces and emojis than for emotion words, suggesting shared neural mechanisms for processing emotional content across these stimulus types.

Complementing these findings, behavioral and physiological studies demonstrate that emojis can elicit affective and autonomic responses comparable to real faces. For example, Gantiva et al. (2021) have shown that happy, angry, and neutral emojis evoke zygomatic muscle activity and skin conductance responses akin to human faces, inducing similar patterns of pleasant and unpleasant affective states. Behavioral results are mixed: Recent studies by Kaye and colleagues explored the emotional and associative properties of emojis through behavioral paradigms. In an Emoji Spatial Stroop Task, Kaye et al. (2022) observed congruency effects for positive emojis presented in upper vertical spaces and negative emojis in lower spaces, suggesting that emojis may carry explicit emotional valence tied to spatial positions. However, follow‐up work (Kaye, MacKenzie, et al. 2023) revealed no implicit effects of emoji valence on response accuracy or latency. Further investigations (Kaye, Rocabado, et al. 2023) demonstrated no facilitative effects of emojis on word processing or memory recall, with emojis failing to enhance associative links to emotion concepts. These findings collectively suggest that while emojis may evoke explicit emotional judgments, their role in implicit cognitive or associative processes remains limited.

Taken together, prior work shows that emotional information from human facial expressions is extracted rapidly, with discriminative neural signals emerging around 100 ms. While existing studies suggest that emojis can elicit affective responses and engage face‐ and emotion‐related networks, they also reveal differences in perceptual and representational processing consistent with their schematic and symbolic nature. Behavioral and physiological findings further indicate that emojis support explicit emotional judgments, while their role in implicit emotional processing appears limited. Importantly, most EEG studies have emphasized differences between emoji and real‐face processing rather than testing whether emotional expressions conveyed by these stimulus types rely on shared neural representations. Consequently, it remains to be tested whether, and to what extent, emotion‐related neural patterns generalize across naturalistic and symbolic facial expressions.

In our previous study using real human faces as stimuli (Ely and Ambrus 2025), we found significant and robust effects associated with facial expression processing, emerging as early as 120 ms and peaking around 160 ms. These findings, consistent across time‐resolved decoding and representational similarity analyses, persisted even when controlling for low‐ and high‐level image properties, suggesting that the observed emotional expression effects are independent of visual image characteristics. This raises the intriguing possibility that face‐like stimuli, such as emojis, may elicit comparable neural patterns in response to emotional expressions.

To test this hypothesis, our study employs multivariate pattern analysis (MVPA) on EEG data to investigate the neural dynamics of facial expression processing across the two distinct stimulus types: real human faces and emoji faces, to determine whether the neural patterns associated with facial expressions are consistent and transferable between these different representations. MVPA leverages distributed patterns of neural activity across sensors and time points to determine whether the neural signal contains information that reliably distinguishes experimental conditions (different facial expressions, in this case). Unlike traditional univariate ERP approaches, which focus on amplitude differences at predefined components and electrodes, MVPA is agnostic to specific peaks and can capture information encoded in spatially and temporally distributed neural patterns that may not be detectable with univariate analyses (Grootswagers et al. 2017). Crucially, beyond assessing decoding performance within each stimulus type, we applied cross‐dataset MVPA by training classifiers on one dataset (e.g., real faces) and testing them on the other (e.g., emoji faces). This approach provides a test of whether the neural representations underlying facial expression processing overlap across different participants and visual formats. Successful cross‐dataset classification indicates that the discriminative neural patterns generalize across stimulus domains, reflecting a transferable neural code rather than stimulus‐specific visual features (Kaplan et al. 2015). We therefore hypothesized that if the neural encoding of facial expressions is abstract and robust, it should be possible to decode displays of emotion not only within each experiment but also across the two experiments. Such findings would suggest that the brain utilizes an overlapping neural code for emotion recognition, irrespective of the visual form of the facial stimulus.

## 2 Materials and Methods

### 2.1 Datasets

Datasets from two experiments were used, one for real human faces, one for emoji faces. The real faces dataset was taken from Ely and Ambrus (2025), the emoji dataset is novel and was acquired for the purposes of this study. The experiments were conducted in accordance with the guidelines of the Declaration of Helsinki. The study was approved by the ethics committee of Bournemouth University [Ethics ID: #52261]. The experiments were designed and implemented using PsychoPy (Peirce 2007; Peirce et al. 2019). Participants in both experiments provided written informed consent before the experiment and took part in the study for partial course credits or volunteered their time. Participants disclosed no history of neurological conditions, had normal or corrected‐to‐normal vision, had not reported taking CNS‐acting medication, and were right‐handed.

### 2.2 Experimental Design

#### 2.2.1 Real Faces Experiment

The stimuli comprised frontal color photographs of eight individuals selected from the KDEF database (Lundqvist et al. 1998). It included four male faces (AM10, AM17, AM24, AM31) and four female faces (AF07, AF15, AF26, AF28), each displaying one of four posed facial expressions: happy, angry, sad, and neutral. This resulted in a total of 32 unique images, which were presented in a randomized sequence.

**FIGURE 1:**
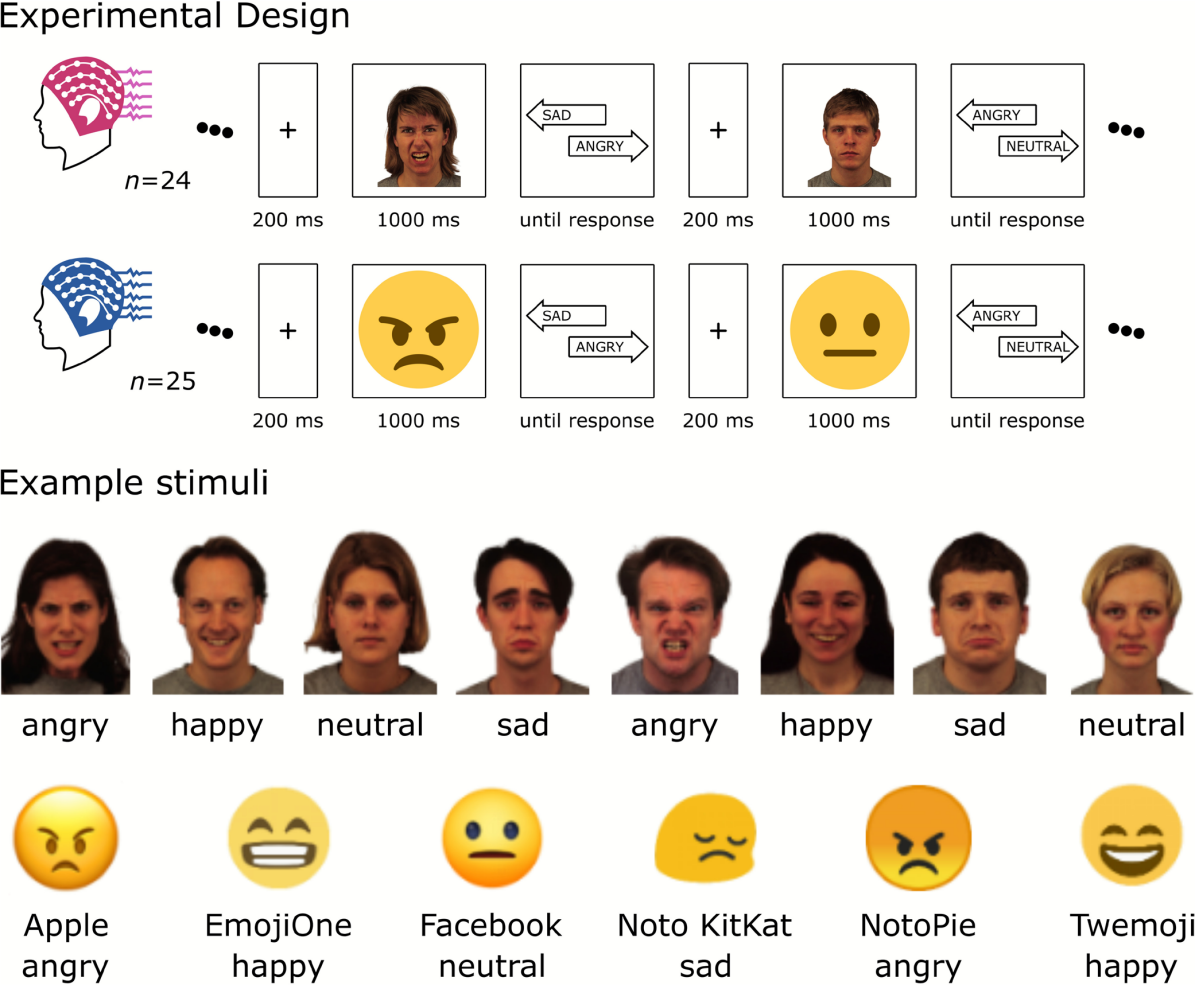
Experimental design and example stimuli. Two experiments were conducted to investigate emotion recognition using a two‐alternative forced choice (2AFC) design. The first study (*n* = 24, reported in Ely and Ambrus 2025) used frontal color photographs of eight individuals (four male, four female) from the KDEF database, each displaying one of four facial expressions—happy, angry, sad, and neutral—resulting in 32 unique images. The second study (*n* = 25) used 24 emoji images sourced from six platforms (Apple, Emoji One, Facebook, Noto KitKat, Noto Pie, and Twemoji) depicting the same four expressions. In both experiments, each trial began with a 200‐ms fixation cross, followed by a 1000‐ms stimulus presentation, and a choice screen displaying one correct and one incorrect emotion label, with an interstimulus interval between 500 and 1000 ms. Participants selected the correct emotion using a keypress with no time limit. For permission to use sample images (permission to display sample stimuli from the KDEF: “To the publisher: Researchers may always include sample images from KDEF or AKDEF in his/her manuscript when said manuscript is a doctoral thesis OR is a manuscript submitted to a scientific journal.” https://kdef.se/faq/using‐and‐publishing‐kdef‐and‐akdef). The image elements are not to scale.

In a two‐alternative forced choice (2AFC) paradigm, each trial began with the display of a fixation cross for 200 ms, followed by the presentation of a face image for 1000 ms. Afterward, a choice screen appeared, presenting one correct and one incorrect emotion label. The interstimulus interval varied between 500 and 1000 ms (Figure 1). Participants (*n* = 24, 8 male, age: 21.21 ± 4.17 years) were tasked with identifying the emotion conveyed by the face by pressing either the left or right key, with no time constraints imposed on their decision. Each face image was shown 12 times. For each presentation, the accurate facial expression was paired with an incorrect option four times, with response key assignments counterbalanced. To maintain an equal number of correct trials, incorrect responses triggered the reinsertion of the trial into the sequence for later presentation. Consequently, the dataset for each participant included 12 repetitions per image, 96 presentations per facial expression, and 48 presentations per identity, summing to a total of 384 trials.

#### 2.2.2 Emoji Faces Experiment

The design of the emoji experiment followed that of the real faces study. The stimuli comprised color emoji images sourced from six platforms: Apple, Emoji One, Facebook, Noto KitKat, Noto Pie, and Twemoji. Each emoji depicted one of four facial expressions—happy, angry, sad, and neutral—resulting in a total of 24 unique images. These images were presented in a randomized sequence.

The experiment employed a two‐alternative forced choice design. Each trial began with a fixation cross displayed for 200 ms, followed by the presentation of an emoji image for 1000 ms. Subsequently, a choice screen appeared, presenting one correct and one incorrect emotion label, with an interstimulus interval ranging from 500 to 1000 ms (Figure 1). Participants (*n* = 25, 5 male, age: 21.08 ± 4.68 years) were instructed to identify the correct emotion depicted by the emoji by pressing the left or right key, with no time constraints on their response.

Each image was repeated four times, with the veridical facial expression paired with an incorrect option for response key assignment across trials to ensure counterbalancing. Incorrect responses prompted the reinsertion of the trial into the sequence for later presentation, ensuring an equal number of correct trials. As a result, each participant's dataset included 12 repetitions per image, 72 presentations per facial expression, and 48 presentations per emoji platform, totaling 288 trials.

### 2.3 EEG Recording and Processing

EEG data were recorded in the EEG laboratory of the Department of Psychology at Bournemouth University using a 64‐channel BioSemi Active‐Two device, with electrode placement based on the international 10‐20 system. Data were acquired at a sampling rate of 2048 Hz using the BioSemi “zero‐reference,” Common Mode Sense (CMS) and Driven Right Leg (DRL) configuration, re‐referenced to the common average offline. The recorded data were processed using MNE‐Python (Gramfort et al. 2013, 2014). Preprocessing included bandpass filtering between 0.1 and 40 Hz, segmentation from −200 to 1200 ms relative to stimulus onset, baseline correction using the 200 ms preceding stimulus presentation, and down‐sampling to 200 Hz. To improve the signal‐to‐noise ratio and optimize computation (Grootswagers et al. 2017), evoked responses for trials with the same image were grouped into bins of three and averaged for each participant. No additional preprocessing steps were applied, as multivariate classifiers can tolerate a degree of noise and residual artifacts (Grootswagers et al. 2017); limiting preprocessing was therefore intended to reduce the risk of attenuating potentially informative signal components and to help preserve statistical power (Carlson et al. 2020; Delorme 2023). A stimulus presentation duration of 1000 ms was selected as a compromise among prior EEG facial expression studies employing shorter exposures (e.g., 200 ms: Balconi and Mazza 2010; Li, Zhang, et al. 2022; Meeren et al. 2005, 500 ms: Muukkonen et al. 2020; Smith and Smith 2019) and longer durations (e.g., 1000 ms: Zhang et al. 2023; 3000 ms: Mavratzakis et al. 2016; Mehdizadehfar et al. 2020). A −200 to 1200 ms analysis window was used to ensure compatibility with our previous and ongoing EEG decoding work (Dalski, Kovács, and Ambrus 2022; Ely and Ambrus 2025; Klink et al. 2023; Ozdemir and Ambrus 2025). Data manipulation was performed using the numpy and scipy packages (Harris et al. 2020; Virtanen et al. 2020).

### 2.4 Analysis Pipelines

Within‐and cross‐dataset classification analyses were carried out. Within‐experiment analyses were conducted to estimate the information content related to the classes of interest for the two stimulus types (emoji and real faces). Cross‐dataset analyses were performed to probe the shared neural signals related to the processing of facial expressions displayed by real and emoji faces. Only correct trials were included. Linear discriminant analysis (LDA) classifiers were systematically trained across participants and datasets to categorize the facial expression, as well as the combinations of facial expressions of the presented stimuli. For a similar approach, see Ambrus (2024).

The within‐dataset analyses employed a leave‐one‐participant‐out approach. For the four‐class emotion classification in the real‐face experiment, the training data included six identities (three male and three female) aggregated from all but one participant, with testing conducted on the left‐out identity in the excluded participant. Similarly, in the emoji experiment, training utilized data from all but one emoji platform aggregated across all but one participant, while testing was performed on the excluded platform in the left‐out participant. The classification of emotional expression pairs followed a similar approach, with the key difference being that only two emotional expressions were included in each classification procedure. In the real‐face experiment, training was performed on six identities (three male and three female), and testing was conducted on one identity excluded during training. In the emoji experiment, training utilized data for all but one platform, while the omitted platform was iteratively used for testing. These processes were repeated iteratively, ensuring that each identity or platform was excluded once for testing in each participant, and every participant was also left out once for testing within the experimental dataset. For a similar approach, see Klink et al. (2023).

The cross‐experiment analyses were conducted following the procedures outlined in Dalski, Kovács, and Ambrus (2022). Classifiers were trained on aggregated data from one dataset and subsequently tested on each participant individually from the other dataset. This analysis was performed in both directions: from emojis to real faces and from real faces to emojis.

Time‐resolved classification was performed across all electrodes and pre‐defined regions of interest (ROIs), covering six scalp locations distributed along the median (left and right) and coronal (anterior, center, and posterior) planes (Ambrus et al. 2019, 2021).

To further characterize the spatial and temporal distribution of information related to facial expression processing, we employed a spatio‐temporal searchlight analysis in sensor space. In this approach, classification analyses were performed repeatedly on local subsets of electrodes (Ambrus 2024; Dalski et al. 2023). Specifically, each EEG channel was tested separately by training and testing a classifier on data from a small electrode cluster consisting of the target channel and its immediately adjacent neighboring sensors. For each such electrode cluster, classification was conducted in a time‐resolved manner across all time points using the same analysis framework as described above. This procedure yielded, for each channel, a time‐resolved measure of decoding performance that reflects the extent to which locally distributed spatio‐temporal patterns at that sensor location contain information distinguishing the experimental conditions, providing a map of where and when discriminative information is expressed across the scalp. Classification analyses were carried out using scikit‐learn (Pedregosa et al. 2011).

#### 2.4.1 Statistical Analyses

A 35 ms moving average (covering 7 consecutive time points) was applied to all participant‐level classification accuracy data (Ambrus 2024; Ambrus et al. 2019, 2021; Dalski et al. 2023; Klink et al. 2023). Statistical analysis of the results included cluster permutation tests as well as Bayesian statistical methods. Classification accuracies were assessed using two‐sided, one‐sample cluster permutation tests (10,000 iterations) against chance (0.25 in the four‐class facial expression classification, 0.5 in the two‐class facial expression pair analyses), implemented in MNE‐Python. The Bayesian analyses (Teichmann et al. 2022) also employed a two‐sided approach, utilizing a non‐directional whole‐Cauchy prior with medium width (*r* = 0.707) and excluding the interval *δ* = −0.5 to +0.5. Bayes factors were then thresholded, with values greater than 10 considered as strong evidence (Moerel et al. 2022; Wetzels et al. 2011). Bayesian analyses were conducted using the BayesFactor R package (Morey et al. 2015).

## 3 Results

### 3.1 Multivariate Classification

#### 3.1.1 Facial Expressions

**FIGURE 2:**
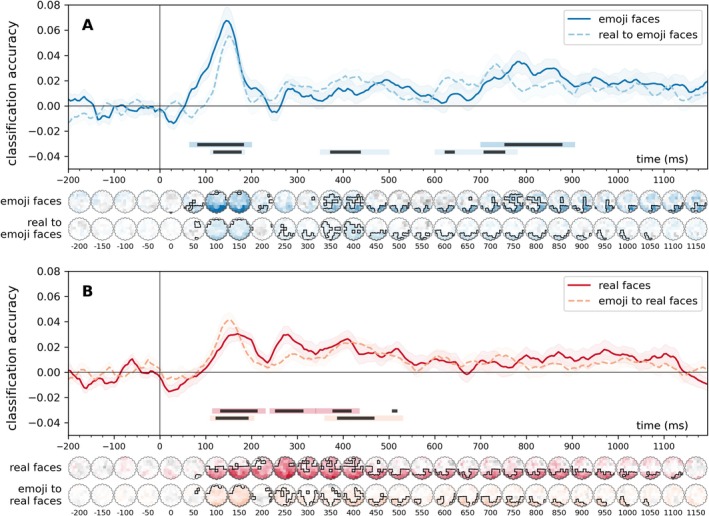
Time‐resolved, within‐experiment (leave‐one‐subject‐out) and cross‐experiment classification of facial expressions, classification accuracies relative to chance (0.25) A: emoji faces and real‐to‐emoji faces, B: real faces and emoji‐to‐real faces. In the within‐experiment analyses (solid lines), classifiers were trained, in a leave‐one‐participant‐out scheme to categorize the facial expressions of the stimuli, separately for the real and the emoji faces datasets. In the cross‐experiment analyses (dashed lines), classifiers were trained on one dataset and were tested on the other. Error ranges represent ±SEM. Light lines denote significant clusters revealed by the two‐sided cluster permutation tests, *p* < 0.05; dark lines denote results of the Bayesian statistical analyses, two‐sided one‐sample Bayesian *t*‐tests, BF > 10, against chance (0.25). Results over all electrodes are presented here. See Supporting Information S1 for the results of the analyses conducted on the pre‐defined regions of interest. Spatio‐temporal searchlight results are shown as scalp maps, with classification accuracy scores averaged in 50‐ms steps. Sensors and time points that belong to significant positive clusters are marked (two‐sided spatio‐temporal cluster permutation tests, *p* < 0.05). For detailed statistics, see Table S1.

Time‐resolved classification of facial expressions (happy, angry, sad, neutral) revealed significant decoding across within‐ and cross‐experiment conditions (Figure 2). For emoji stimuli, two clusters emerged: an early window (70–195 ms, *p* < 0.01, peak Cohen's *d* = 1.21) and a late window (705–900 ms, *p* < 0.01, Cohen's *d* = 0.80). In the real face experiment, three significant clusters were observed between 120 and 430 ms (cluster *p* < 0.05, peak Cohen's *d*s = 0.78–1.20). Cross‐experiment decoding (2AFC to emoji and emoji to 2AFC) yielded early (115–200 ms) and mid‐late (355–525 ms) clusters with robust effect sizes (peak Cohen's *d*s = 0.77–1.56).

Similarly, spatio‐temporal searchlight classification of facial expressions revealed robust decoding in all analyses. For emoji faces, two significant clusters were identified (50–225 ms and 350–1195 ms; cluster *p*s < 0.01) with peak decoding at P6 (peak Cohen's *d* = 1.91) and PO4 (*d* = 1.14). Real faces showed one broad cluster (80–1145 ms; *p* < 0.0001) peaking at PO7 (*d* = 1.65). Cross‐experiment results indicated significant decoding for real‐to‐emoji (90–1065 ms; *p*s < 0.01) and emoji‐to‐real faces (75–1060 ms; *p*s < 0.05) with strong effects across occipital and parietal channels (Cohen's *d*s ≤ 1.0).

#### 3.1.2 Facial Expression Pairs

**FIGURE 3:**
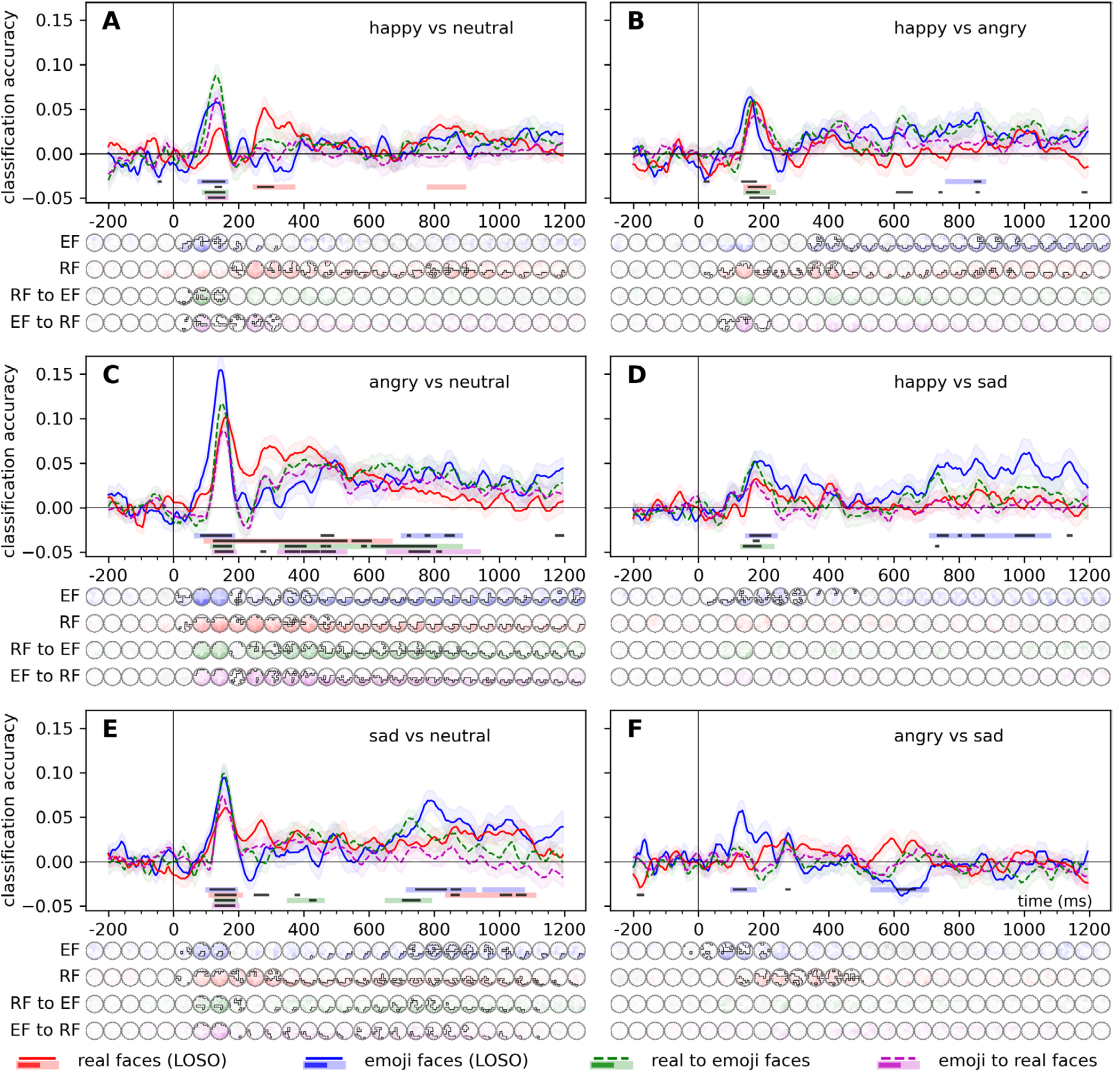
Time‐resolved, within‐experiment (leave‐one‐subject‐out) and cross‐experiment classification of facial expression pairs, classification accuracies relative to chance (0.5) A: happy vs neutral expressions, B: happy vs angry expressions, C: angry vs neutral expressions, D: happy vs sad expressions, E: sad vs neutral expressions, F: angry vs sad expressions. For the within‐experiment classification (solid lines), training was iteratively performed on six identities (3 male and 3 female) and tested on one left out in the real faces experiment, while in the emoji faces experiment, training was iteratively performed on five platforms and tested one platform left out. In the cross‐experiment analyses (dashed lines), classifiers were trained on one dataset and were tested on the other. Error ranges represent ±SEM. Light lines denote significant clusters revealed by the two‐sided cluster permutation tests, *p* < 0.05; dark lines denote results of the Bayesian statistical analyses, two‐sided one‐sample Bayesian *t*‐tests, BF > 10, against chance (0.5). Results over all electrodes are presented here. See Supporting Information S1 for the results of the analyses conducted on the pre‐defined regions of interest. Spatio‐temporal searchlight results are shown as scalp maps, with classification accuracy scores averaged in 50‐ms steps. Sensors and time points that belong to significant positive clusters are marked (two‐sided spatio‐temporal cluster permutation tests, *p* < 0.05). For detailed statistics, see Table S1.

Time‐resolved classification revealed significant decoding of facial expressions across within‐ and cross‐experiment analyses (Figure 3). For real faces, decoding was most robust for angry versus neutral pairs, showing a broad temporal window (100–665 ms, cluster *p* < 0.001, peak Cohen's *d* = 2.11). Other pairs, such as happy versus neutral and sad versus neutral, exhibited narrower windows with positive decoding clusters peaking within 115–280 ms. For emoji faces, early significant decoding was observed for happy versus neutral and angry versus neutral (70–180 ms, Cohen's *d*s > 1.5), with additional late‐stage clusters. Cross‐experiment results confirmed early decoding (95–160 ms, Cohen's *ds* > 1.51) for real‐to‐emoji transfer, particularly for neutral and angry expressions.

Similarly, the spatio‐temporal searchlight analysis revealed robust and significant neural differentiation for various facial expression pairs across the experimental conditions. For real faces, decoding of facial expression pairs such as happy versus neutral, angry versus neutral, and sad versus neutral showed early and sustained differentiation, with clusters typically beginning within 75–215 ms and peaking around 150–290 ms at posterior channels (e.g., POz, PO8), with Cohen's *d* values exceeding 1.6 in some cases. Similarly, for emoji faces, contrasts such as happy vs. neutral and angry vs. neutral demonstrated early neural differentiation, with clusters starting as early as 55 ms and showing multiple clusters of significant decoding throughout the trial, with Cohen's *d* values exceeding 2.1. Cross‐experiment analysis confirmed significant generalization, particularly for angry versus neutral and sad versus neutral pairs, with decoding onset within 85–120 ms.

More detailed results can be found in Table S1.

### 3.2 Effect Onsets

**FIGURE 4:**
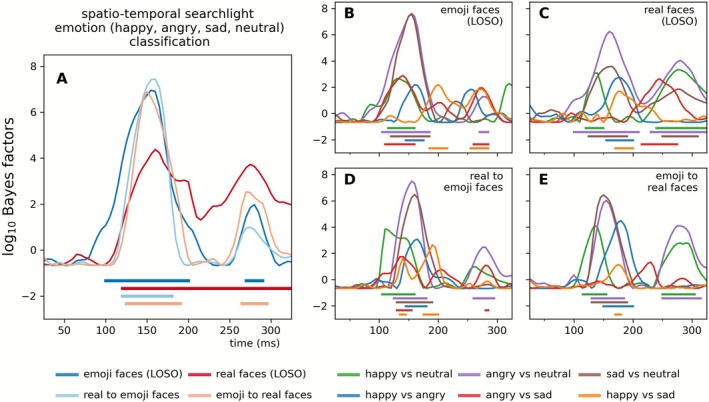
Effect onset estimates. Time‐resolved spatio‐temporal searchlight classification was performed on all channels and their neighboring electrodes. Classification performance was averaged across all sensors to probe the onset of the effects. Onsets were determined by two‐tailed Bayesian *t*‐tests against chance, with BF values exceeding 10 considered indicative of strong evidence (horizontal significance markers). The figure depicts time‐resolved Bayes factors. For classification accuracies, see Figure S3.

To estimate the temporal onset of discriminative information in the EEG signal, we performed a whole‐scalp onset analysis based on time‐resolved searchlight decoding. Decoding accuracies were averaged across all searchlight locations at each time point, resulting in a spatially agnostic measure of when emotional expression information first became reliably decodable (for a similar approach, see Ely and Ambrus 2025). Because this analysis collapses across local electrode clusters, it provides a coarse temporal summary rather than spatially specific information. Detailed time courses and spatial distributions of decoding performance (both within and across stimulus sets) are reported above in the main classification analyses using all‐electrodes and region‐specific approaches. This onset analysis thus serves as a supplementary summary of the temporal dynamics captured by the full decoding results. Figure 4 shows these results for 4‐class facial expression classification (Figure 4A) and classification of emotion pairs (Figure 4B–E). See Table S1 for further details.

Time‐resolved spatio‐temporal searchlight analyses revealed consistent decoding of emotional expressions within and across experiments. For emoji faces in the within‐experiment leave‐one‐subject‐out (LOSO) classification, emotion decoding onset at 100 ms peaked at 155 ms (BF > 10^6^), with pairwise contrasts showing early and robust effects. Happy versus neutral decoding began at 115 ms, peaking at 130 ms (BF = 458.97), while angry versus neutral started at 105 ms and peaked at 155 ms (BF > 10^7^). Sad versus neutral had an onset at 120 ms, peaking similarly at 155 ms (BF > 10^7^). Other contrasts, such as happy versus angry and happy versus sad, showed later onsets and peaks, typically around 145–200 ms.

For real faces, within‐experiment LOSO classification showed an emotion onset at 120 ms with a peak at 160 ms (BF > 10^4^). Pairwise contrasts largely mirrored those seen in emojis, though some contrasts, such as angry vs. sad (onset: 215 ms, peak: 245 ms; BF = 430.02), exhibited later effects.

In cross‐experiment analyses, the four‐class emotion decoding showed comparable temporal profiles across real‐to‐emoji and emoji‐to‐real transfers. Onsets ranged from 120 to 125 ms with peaks at 150–160 ms (BF > 10^6^), confirming shared neural coding of emotional expressions. Pairwise contrasts revealed earlier decoding for real‐to‐emoji classifications, such as happy vs. neutral (onset: 105 ms, peak: 110 ms; BF > 10^3^) and angry vs. neutral (onset: 125 ms, peak: 155 ms; BF > 10^7^). Emoji‐to‐real classification showed later effects, with decoding onset at 115–170 ms and peaks up to 180 ms, though angry vs. sad showed no significant effects.

## 4 Discussion

The present study asked whether the neural information supporting recognition of emotional expressions generalizes between real human faces and face‐like emojis. Using time‐resolved EEG decoding, we observed robust classification of emotional expressions both within each stimulus format, using leave‐one‐subject‐out (LOSO) cross‐validation, and across formats, by training classifiers on one experiment and testing them on the other. Across these analyses, discriminative information emerged early (100–120 ms) and peaked in the N170 time range (145–160 ms) over posterior occipital and parietal sites, with additional overlapping effects in a later window (350–500 ms). These temporal characteristics place the shared signal within canonical stages of face‐sensitive perceptual processing, while also pointing to later evaluative or integrative activity (Bentin et al. 1996; Eimer 2011; Gao et al. 2019).

Within‐experiment LOSO decoding demonstrates that expression‐related neural patterns were reliable across participants within each stimulus class, indicating that the observed effects were not driven by individual‐specific strategies or idiosyncratic responses. Building on this, cross‐experiment classification provided a more stringent test of generalization by assessing whether these expression‐related patterns transferred across both participant groups and stimulus domains (Ambrus 2024; Kaplan et al. 2015). Successful cross‐format decoding therefore suggests that at least part of the neural signal distinguishing emotional expressions is sufficiently stable to generalize beyond specific identities, participants, or low‐level stimulus features. While within‐subject designs offer tighter control over inter‐individual variability, the present combination of LOSO and cross‐experiment decoding emphasizes robustness at the population level and allows stronger inferences about shared representational structure across naturalistic and symbolic facial expressions.

In this context, our results show that emojis, despite their schematic and symbolic nature, engage early EEG codes that are comparable in timing and scalp distribution to those elicited by photographic faces. This finding aligns with previous work suggesting that simplified face‐like stimuli can recruit similar perceptual mechanisms to real faces, particularly within the N170 time window (Henderson et al. 2003). At the level of information content, expression‐related neural patterns were broadly similar across formats, but subtle differences were observed: emojis showed more pronounced pairwise separability among happy, angry, sad, and neutral expressions, plausibly reflecting their schematic and exaggerated designs, which may amplify morphological distinctiveness (e.g., mouth curvature, brow shape, eye configuration), rendering category boundaries visually stark and readily decodable at face‐sensitive latencies. Previous behavioral and EEG work likewise notes advantages for emojis over photographs in speed and clarity of emotional signaling (Kaye et al. 2021; Dalle Nogare and Proverbio 2023). In contrast, real faces are subtler and more variable across identity, pose, and texture; consistent with this, within‐valence separations (e.g., angry vs. sad) were weaker early on, mirroring behavioral patterns, where differentiating between negative emotional expressions (fearful, disgusted, sad, angry) is found to be more challenging (Calvo and Beltrán 2013; Zhang et al. 2023).

One interpretation of our results, consistent with the broader affect literature, is that early decoding in the N170 window may reflect coarse affective properties (e.g., valence/emotional salience), with finer‐grained categorical distinctions becoming more pronounced later or under conditions that enhance perceptual distinctiveness (Farkas and Sabatinelli 2023; Grootswagers et al. 2020; Lindquist et al. 2016). However, our data do not compel a strictly valence‐first conclusion. The meta‐analytic literature indicates that the N170 can show heterogeneous sensitivity across emotions, with anger (and fear/happiness) sometimes eliciting larger modulation than other categories (Hinojosa et al. 2015). For affectively charged scenes, Groottswagers and colleagues (2020) have demonstrated that valence and arousal as well as discrete emotions were differentiable from the MEG response, with several emotional constructs represented early and distinctly. It is therefore plausible that an early, partly category‐specific code exists, especially when diagnostic features are salient, and that emojis simply magnify such diagnostic cues relative to real faces. In short, while valence‐related structure is a parsimonious account of the broad pattern, an early distinct representation remains compatible with our findings and the literature.

Beyond the early window, we observed cross‐format overlap in the 350–500 ms range. Whereas early decoding aligns with face‐sensitive perceptual encoding, effects in this later window plausibly index supramodal evaluative processes, for example, appraisal and decision‐related signals less tied to visual format and more related to task demands and affective (Bo et al. 2022; Farkas and Sabatinelli 2023). Converging MVPA evidence from fMRI identifies format‐independent emotion representations in medial prefrontal cortex and superior temporal sulcus, consistent with abstract, modality‐independent codes that become more apparent as processing unfolds (Klasen et al. 2011; Peelen et al. 2010). Meta‐analytic work also suggests that valence is flexibly implemented across valence‐general limbic/paralimbic regions rather than a single bipolar system, again, compatible with later integrative processing (Lindquist et al. 2016).

Taken together, the data can be accommodated by two complementary views. A face‐specific account emphasizes that emojis and photographs tap shared perceptual face‐sensitive pathways in the N170 range (Bentin et al. 1996; Gao et al. 2019), with early decodable information reflecting a mixture of emotion category‐specific diagnostic features and coarse affective properties. A supramodal account highlights the cross‐format generalization and the mid‐late overlap as signatures of abstract, format‐independent emotion codes in higher‐order networks (Klasen et al. 2011; Lindquist et al. 2016; Peelen et al. 2010).

The scope of this study was intentionally stimulus‐locked and timing‐focused: we asked when EEG patterns carry information sufficient to discriminate emotional expressions across two visual face formats under an explicit recognition task. The stimulus sets were controlled (posed identities from a single database; standardized emojis), appropriate for isolating temporal dynamics and format generalization but not for capturing the full variability of spontaneous or contextually embedded expressions. Accordingly, we do not claim to adjudicate socio‐cognitive influences (e.g., semantics or goals), nor do we assert that arousal and valence uniformly dominates early encoding across all contexts, nor do we imply that naturalistic expression processing is reducible to schematic depictions; rather, it shows that both formats engage overlapping representational structure sufficient for discrimination under the present task. Future cross‐classification work should broaden real‐face stimuli (diverse identities, spontaneous expressions, head poses), extend cross‐stimulus designs to voices, bodies, scenes, and words to probe supramodal generality, and orthogonally manipulate context and social priors to determine how and when discrete categories, if present, emerge over and above coarse affect in time.

## 5 Future Directions

Our study opens several promising avenues for further research into the neural mechanisms underlying affective processing. One important direction is to extend the range of emotional expressions examined, moving beyond basic categories such as happiness, sadness, anger, and neutrality to include complex or mixed emotions. This is especially relevant in aiding the design of interactive affective systems and instruments aiming to reduce linguistic confounds (Kaye and Schweiger 2023).

To disentangle the role of exposure‐dependent learning, future work could investigate developmental cohorts: early sensitivity to emojis as faces in infants or young children with minimal cultural exposure would suggest innate biases, while delayed emergence would point to learned associations. Similarly, testing older adults who first encountered emojis after critical periods for face perception could reveal whether cross‐classification diminishes with late exposure, implicating a more learning‐dependent effect. Additionally, employing novel, culturally unfamiliar symbols—such as artificial emojis or platform‐specific designs absent from participants' prior experience—could clarify whether preserved cross‐decoding relies on evolved mechanisms for schematic face‐like patterns.

Complementing these investigations, cross‐cultural studies could further explore whether the neural correspondence between emojis and real faces reflects universal mechanisms or culturally mediated processes. The comparison of neural responses to emotional expressions across populations with differing norms in emoji usage or interpretation could help us assess the universality of shared spatio‐temporal codes. For example, cultures that attribute distinct meanings to specific emojis may exhibit reduced cross‐classification for culturally ambiguous symbols. Conversely, preserved neural overlap for universally recognized emotions (e.g., happiness) across cultures would strengthen claims of evolved, domain‐general mechanisms. Such work would not only clarify the global applicability of these findings but also identify cultural biases in how symbolic representations engage neural systems, bridging gaps between biological predispositions and sociocultural influences on emotion processing.

## 6 Strengths of the Current Approach

The methodological framework of this study offers several key strengths. First, multivariate pattern analysis to EEG data provides unparalleled flexibility in probing shared neural representations across distinct stimulus types (Ambrus 2024; Dalski, Kovács, and Ambrus 2022, 2023; Dalski, Kovács, Wiese, and Ambrus 2022; Klink et al. 2023; Li, Burton, et al. 2022; Ozdemir and Ambrus 2025). By training classifiers on one dataset (e.g., real faces) and testing on another (e.g., emojis) we isolated emotion‐specific neural codes independent of low‐level visual features. This approach inherently controlled for image properties (e.g., color, luminance, spatial frequency), as real faces and emojis differ markedly in their visual characteristics but converge in their emotional content. The use of identical experimental paradigms across the two experiments further minimized confounds related to the task.

Second, the study's design is highly generalizable. Training classifiers on one dataset and testing them on another provides a stringent generalization test that goes beyond within‐subject comparisons; the analytic framework is expressly suited to cross‐dataset generalization and has been applied effectively across heterogeneous EEG datasets with differing trial counts (Ambrus 2024), tasks, devices (Dalski, Kovács, Wiese, and Ambrus 2022; Li, Burton, et al. 2022), sample sizes and populations (Xie et al. 2022). Successful cross‐dataset decoding indicates that discriminative neural patterns are sufficiently stable to transfer across different stimulus sets and participant groups, rather than being tied to idiosyncratic visual features or subject‐specific response characteristics. Although a within‐subject design would control for inter‐individual variability, the present between‐dataset approach allowed us to demonstrate robustness and generalizability of neural representations across populations and stimulus domains. The bidirectional cross‐classification framework (Kaplan et al. 2015), here, testing both real‐to‐emoji and emoji‐to‐real decoding, provides a strong template for future research. For example, this approach could be extended to developmental cohorts to investigate how neural representations of symbolic stimuli emerge with age, or to cross‐cultural studies comparing populations with divergent emoji interpretation norms. Similarly, clinical populations (e.g., individuals with autism spectrum disorder or prosopagnosia) could be tested to determine whether atypical face‐processing mechanisms extend to schematic stimuli like emojis.

The data‐driven nature of MVPA, free from a priori assumptions about regions or components of interest, enables the discovery of unanticipated patterns. This methodological agnosticism is particularly advantageous for studying novel stimuli such as emojis, where established neural markers may not fully apply (Carlson et al. 2020; Grootswagers et al. 2017).

## 7 Limitations

The use of multivariate pattern analysis on EEG data, combined with bidirectional cross‐classification between stimulus types, provided a stringent test of shared emotion‐specific neural representations while controlling for low‐level visual differences. This framework has proven robust when generalizing across heterogeneous datasets with differing trial numbers, tasks, and participant characteristics (Ambrus 2024; Dalski et al. 2023; Dalski, Kovács, Wiese, and Ambrus 2022; Xie et al. 2022).

Several limitations should nevertheless be noted. The real‐faces experiment contained more trials per expression (96 presentations, 384 trials total) than the emoji experiment (72 presentations, 288 trials total). While MVPA is relatively tolerant of moderate differences in trial count and successful cross‐dataset generalization has been demonstrated across unequal trial numbers in prior work, the lower number of emoji trials may have modestly reduced classifier stability or statistical power for that condition. This design choice reflected the effort to balance signal‐to‐noise ratios across stimulus types: real‐face stimuli inherently exhibit greater low‐level visual variability (e.g., in luminance, texture, and facial identities), necessitating more trials to achieve reliable MVPA decoding, whereas schematic emojis are more uniform and have been shown to elicit more consistent behavioral recognition (e.g., higher accuracy and faster response times; Dalle Nogare and Proverbio 2023). We reasoned that equating trial counts exactly would have required either reducing real‐face trials (potentially compromising decoding reliability) or extending the emoji session, increasing participant burden. The resulting longer duration for the real‐faces experiment raises the possibility of greater fatigue effects in that experiment, though participants were given self‐paced breaks as needed in both experiments.

The present samples are modest in size and, in both experiments, predominantly female. Although MVPA approaches can yield reliable decoding with sample sizes comparable to those used here, and cross‐experiment generalization helps mitigate concerns about dataset‐specific effects, future studies with larger and more gender‐balanced samples will be important for evaluating the generalizability of these findings. Previous work has reported small average differences in facial expression recognition performance between sexes (Thompson and Voyer 2014); however, the current study was not designed to assess such effects. As the present design was not powered to detect subtle sex differences in expression processing, dedicated investigations with sufficient power and balanced designs will be necessary to determine whether and how sex‐related factors influence the neural representations of facial expressions observed here.

## 8 Summary

This study investigated the neural dynamics underlying emotion recognition in emoji faces and contrasted it with that of real human faces, using EEG and multivariate pattern analysis. Across two experiments, participants performed emotion classification tasks while viewing happy, angry, sad, and neutral expressions in real faces or emojis. Robust decoding of emotional expressions emerged as early as 100–120 ms, peaking at 145–160 ms over posterior‐occipital and parietal regions, with spatio‐temporal patterns mirroring the N170 component—a hallmark of face‐specific processing. Effects between the 350–500 ms post‐stimulus time window were also observed. Critically, cross‐classification analyses revealed bidirectional generalization of neural codes between real and emoji faces, particularly for emotion pairs involving neutral expressions (e.g., angry vs. neutral). This suggests that schematic emojis engage neural mechanisms that overlap with those for facial expression processing, despite their artificiality. These findings advance our understanding of how symbolic and naturalistic social signals are processed, demonstrating the brain's flexibility in the interpretation of abstract emotional cues. Our findings add to the growing body of work in semiotics that explores how humans assign emotional and semantic meaning to symbolic objects (Logi and Zappavigna 2023), emphasizing the importance of emojis as more than mere decorative elements in communication. The results hold implications for digital communication, where emojis serve as proxies for facial expressions, and for designing emotionally responsive technologies that align with human social cognition. Future work should explore the extent of evolved and learned contributions to this overlap, including cross‐cultural variability in emoji interpretation and developmental trajectories of symbolic emotion processing. This research further demonstrates the adaptability of human social perception in an increasingly online world.

## Author Contributions

**Madeline Molly Ely:** conceptualization, investigation, writing – original draft, writing – review and editing, formal analysis, methodology. **Chloe Kelsey:** conceptualization, investigation, writing – review and editing, writing – original draft, formal analysis. **Géza Gergely Ambrus:** conceptualization, investigation, methodology, software, data curation, supervision, writing – original draft, writing – review and editing, formal analysis, visualization.

## Funding

The authors have nothing to report.

## Conflicts of Interest

The authors declare no conflicts of interest.

## Supporting information


**Table S1:** Detailed results of the statistical analysis in tabular form.
**Figure S1:** Time‐resolved, within‐experiment (leave‐one‐subject‐out) and cross‐experiment classification of facial expressions. In the within‐experiment analyses (LOSO), classifiers were trained, in a leave‐one‐participant‐out scheme to categorize the facial expressions of the stimuli, separately for the real and the emoji faces datasets. In the cross‐experiment analyses, classifiers were trained on one dataset and were tested on the other. Error ranges represent ±SEM. Light lines denote significant clusters revealed by the two‐sided cluster permutation tests, p < 0.05; dark lines denote results of the Bayesian statistical analyses, two‐sided one‐sample Bayesian t‐tests, BF > 10, against chance (0.25). Results over all electrodes and pre‐defined regions of interest are presented here. For detailed statistics, see Table S1. Supplements Figure 3 in the main text.
**Figure S2:** Time‐resolved, within‐experiment (leave‐one‐subject‐out) and cross‐experiment classification of facial expression pairs. For the within‐experiment classification (LOSO), training was iteratively performed on six identities (3 male and 3 female) and tested on one left out in the real faces experiment, while in the emoji faces experiment, training was iteratively performed on five platforms and tested one platform left out. In the cross‐experiment analyses, classifiers were trained on one dataset and were tested on the other. Error ranges represent ±SEM. Light lines denote significant clusters revealed by the two‐sided cluster permutation tests, p < 0.05; dark lines denote results of the Bayesian statistical analyses, two‐sided one‐sample Bayesian t‐tests, BF > 10, against chance (0.5). Results over all electrodes and pre‐defined regions of interest are presented here. For detailed statistics, see Table S1. Supplements Figure 4 in the main text.
**Figure S3:** Spatiotemporal searchlight classification accuracies over all electrodes. Time‐resolved spatio‐temporal searchlight classification was performed on all channels and their neighboring electrodes. Classification performance was averaged across all sensors. Dark significance markers represent two‐tailed Bayesian t‐tests against chance, with BF values exceeding 10 considered indicative of strong evidence (horizontal significance markers). Light lines denote significant clusters revealed by the two‐sided cluster permutation tests, p < 0.05. Supplements Figure 2 in the main text.

## Data Availability

The data that support the findings of this study are openly available in OSF at https://osf.io, reference number https://osf.io/4ctnp/.
