# Supplementary material for: Shared Neural Codes for Emotion Recognition in Emoji and Human Faces
Source: Psychophysiology. 2026 Mar 2;63(3):e70268. doi: 10.1111/psyp.70268 (PMC12954366; doi:10.1111/psyp.70268)
Supplement: Supplementary file 1 — Table S1: Detailed results of the statistical analysis in tabular form. Figure S1: Time‐resolved, within‐experiment (leave‐one‐subject‐out) and cross‐experiment classification of facial expressions. In the within‐experiment analyses (LOSO), classifiers were trained, in a leave‐one‐participant‐out scheme to categorize the facial expressions of the stimuli, separately for the real and the emoji faces datasets. In the cross‐experiment analyses, classifiers were trained on one dataset and were tested on the other. Error ranges represent ±SEM. Light lines denote significant clusters revealed by the two‐sided cluster permutation tests, p < 0.05; dark lines denote results of the Bayesian statistical analyses, two‐sided one‐sample Bayesian t‐tests, BF > 10, against chance (0.25). Results over all electrodes and pre‐defined regions of interest are presented here. For detailed statistics, see Table S1. Supplements Figure 3 in the main text. Figure S2: Time‐resolved, within‐experiment (leave‐one‐subject‐out) and cross‐experiment classification of facial expression pairs. For the within‐experiment classification (LOSO), training was iteratively performed on six identities (3 male and 3 female) and tested on one left out in the real faces experiment, while in the emoji faces experiment, training was iteratively performed on five platforms and tested one platform left out. In the cross‐experiment analyses, classifiers were trained on one dataset and were tested on the other. Error ranges represent ±SEM. Light lines denote significant clusters revealed by the two‐sided cluster permutation tests, p < 0.05; dark lines denote results of the Bayesian statistical analyses, two‐sided one‐sample Bayesian t‐tests, BF > 10, against chance (0.5). Results over all electrodes and pre‐defined regions of interest are presented here. For detailed statistics, see Table S1. Supplements Figure 4 in the main text. Figure S3: Spatiotemporal searchlight classification accuracies over all electrodes. T [file PSYP-63-e70268-s001.zip › psyp70268-sup-0002-Supinfo1@Supplementary_Information.docx]

# Supplementary Information

**Shared Neural Codes for Emotion Recognition in Emoji and Human Faces**

**Supplementary Information 1**

**Multivariate classification**

| **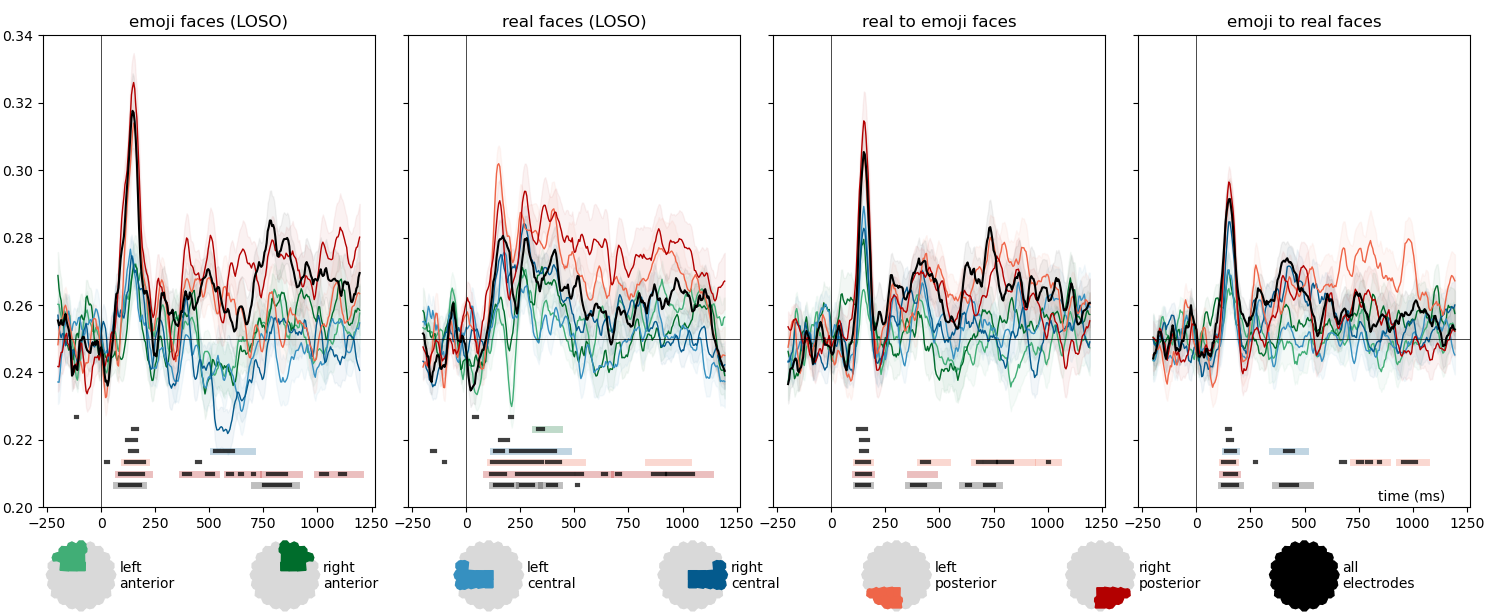** |
| --- |
| **Supplementary Information Figure 1. Time-resolved, within-experiment (leave-one-subject-out) and cross-experiment classification of facial expressions.** In the within-experiment analyses (LOSO), classifiers were trained, in a leave-one-participant-out scheme to categorize the facial expressions of the stimuli, separately for the real and the emoji faces datasets. In the cross-experiment analyses, classifiers were trained on one dataset and were tested on the other. Error ranges represent ±SEM. Light lines denote significant clusters revealed by the two-sided cluster permutation tests,*p*< 0.05; dark lines denote results of the Bayesian statistical analyses, two-sided one-sample Bayesian *t*-tests, BF > 10, against chance (0.25). Results over all electrodes and pre-defined regions of interest are presented here. For detailed statistics, see **Supplementary Table 1**. Supplements **Figure 3** in the main text. |

| 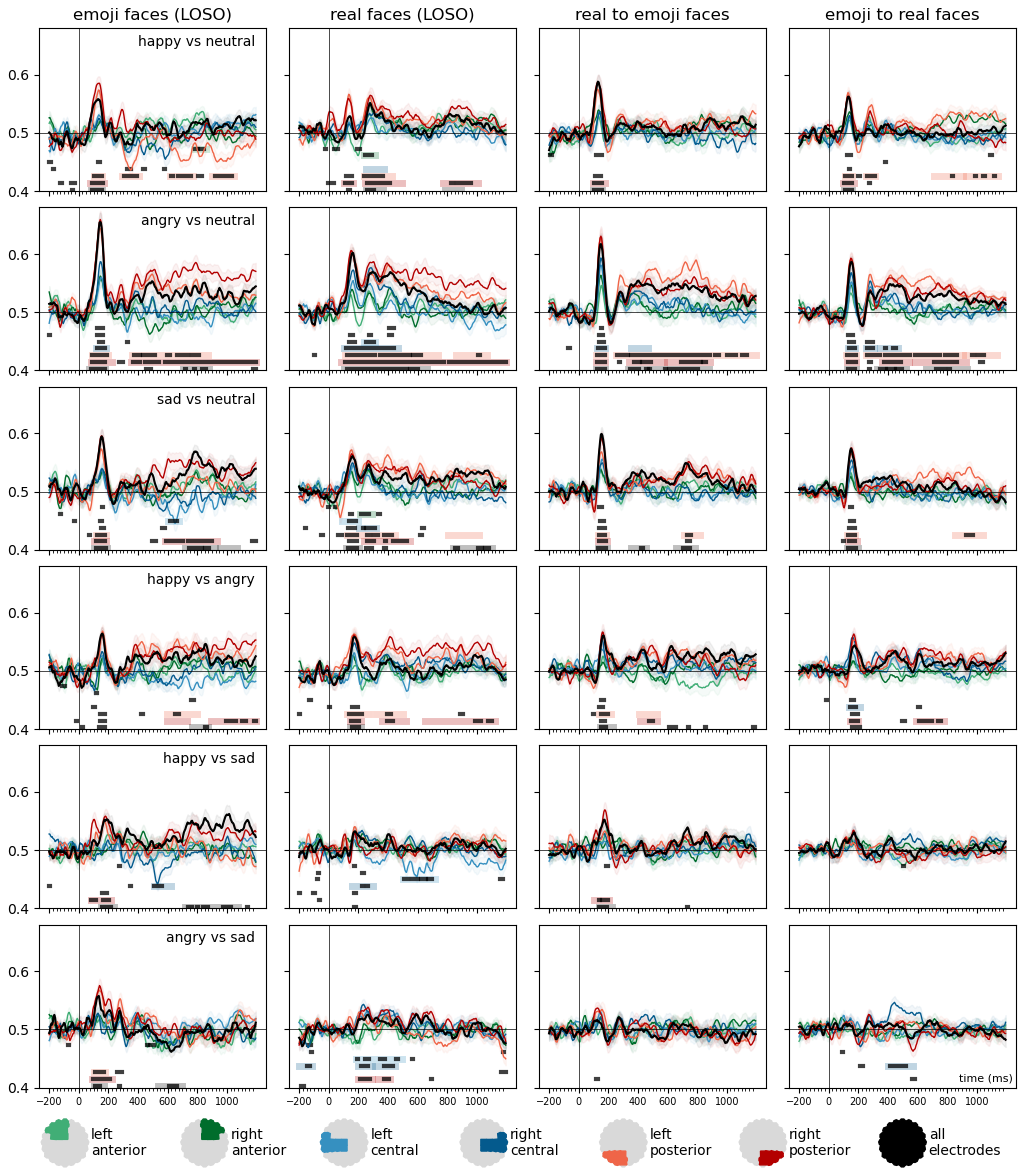 |
| --- |
| **Supplementary Information Figure 2. Time-resolved, within-experiment (leave-one-subject-out) and cross-experiment classification of facial expression pairs.** For the within-experiment classification (LOSO), training was iteratively performed on six identities (3 male and 3 female) and tested on one left out in the real faces experiment, while in the emoji faces experiment, training was iteratively performed on five platforms and tested one platform left out. In the cross-experiment analyses, classifiers were trained on one dataset and were tested on the other. Error ranges represent ±SEM. Light lines denote significant clusters revealed by the two-sided cluster permutation tests,*p*< 0.05; dark lines denote results of the Bayesian statistical analyses, two-sided one-sample Bayesian *t*-tests, BF > 10, against chance (0.5). Results over all electrodes and pre-defined regions of interest are presented here. For detailed statistics, see **Supplementary Table 1**. Supplements **Figure 4** in the main text. |

**Supplementary Information 2**

**Effect onsets**

|  |  | within-experiment | | cross-experiment | |
| --- | --- | --- | --- | --- | --- |
|  |  | emoji  faces | real  faces | real to  emoji faces | emoji to  real faces |
| emotion | onset | 100 ms | 120 ms | 120 ms | 125 ms |
| 4-class | peak | 155 ms | 160 ms | 160 ms | 150 ms |
|  | peak BF | >10^6^ | >10^4^ | >10^7^ | >10^6^ |
| happy vs neutral | onset | 115 ms | 120 ms | 105 ms | 115 ms |
|  | peak | 130 ms | 135 ms | 110 ms | 135 ms |
|  | peak BF | 458.97 | >10^3^ | >10^3^ | >10^4^ |
| angry vs neutral | onset | 105 ms | 100 ms | 125 ms | 130 ms |
|  | peak | 155 ms | 160 ms | 155 ms | 155 ms |
|  | peak BF | >10^7^ | >10^6^ | >10^7^ | >10^6^ |
| sad vs neutral | onset | 120 ms | 125 ms | 130 ms | 130 ms |
|  | peak | 155 ms | 165 ms | 160 ms | 150 ms |
|  | peak BF | >10^7^ | >10^3^ | >10^6^ | >10^6^ |
| happy vs angry | onset | 145 ms | 155 ms | 145 ms | 150 ms |
|  | peak | 160 ms | 175 ms | 165 ms | 180 ms |
|  | peak BF | 156.02 | 543.72 | >10^3^ | >10^4^ |
| angry vs sad | onset | 110 ms | 215 ms | 130 ms | - |
|  | peak | 140 ms | 245 ms | 140 ms | - |
|  | peak BF | 782.21 | 430.02 | 57.1 | - |
| happy vs sad | onset | 185 ms | 170 ms | 135 ms | 170 ms |
|  | peak | 200 ms | 175 ms | 140 ms | 175 ms |
|  | peak BF | 155.56 | 49.46 | 41.68 | 13.9 |

**Supplementary Information Table 1. Onsets and peaks.** Time-resolved spatio-temporal searchlight classification averaged across all sensors. Onsets were determined by two-tailed Bayesian *t*‑tests against chance, with BF values exceeding 10 considered indicative of strong evidence. Supplements **Figure 2** in the main text.

| 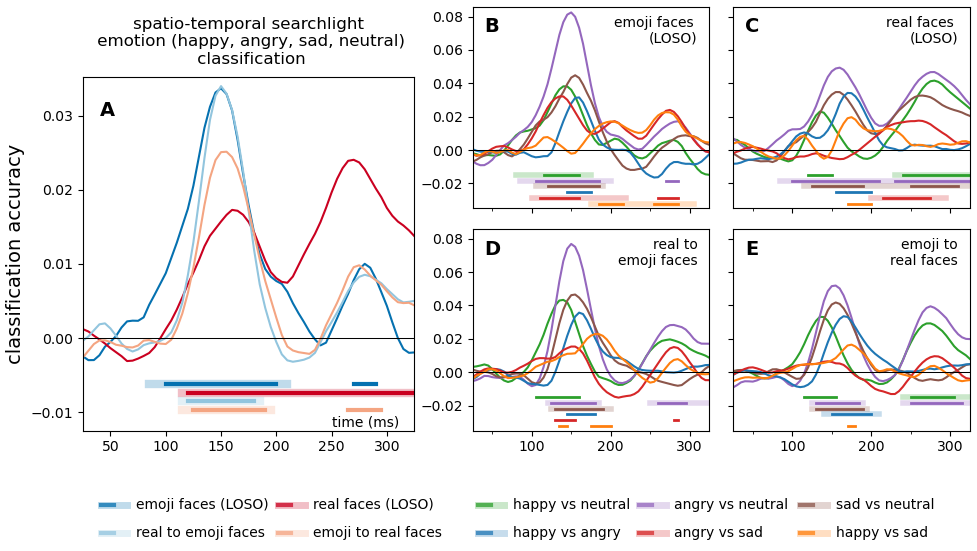 |
| --- |
| **Supplementary Information Figure 3. Spatiotemporal searchlight classification accuracies over all electrodes.** Time-resolved spatio-temporal searchlight classification was performed on all channels and their neighboring electrodes. Classification performance was averaged across all sensors. Dark significance markers represent two-tailed Bayesian *t*-tests against chance, with BF values exceeding 10 considered indicative of strong evidence (horizontal significance markers). Light lines denote significant clusters revealed by the two-sided cluster permutation tests, *p* < 0.05. Supplements **Figure 2** in the main text. |
